# Validation of a Modular Base Editing Strategy, Free‐BE, in *Physcomitrium patens*, Maize, and Wheat

**DOI:** 10.1002/pld3.70196

**Published:** 2026-10-15

**Authors:** Anouchka Guyon‐Debast, Marie Javelle, Pierre‐François Perroud, Jean‐Philippe Pichon, Wyatt Paul, Fabien Nogué

**Affiliations:** ^1^ Université Paris‐Saclay, INRAE, AgroParisTech Institute Jean‐Pierre Bourgin for Plant Sciences Versailles France; ^2^ Limagrain Field Seeds Centre de Recherche de Chappes Chappes France

**Keywords:** base editing, CRISPR, Free‐BE, maize, *Physcomitrium patens*, wheat

## Abstract

Base editing (BE) technologies enable precise genome modification but often generate undesired editing outcomes, including indels, bystander edits, and editing outside the optimal activity windows. Here, we report a modular BE strategy, termed Free‐BE, where the cytidine deaminase and Cas9 nickase are delivered as separate components rather than as a single fusion protein. In the moss *Physcomitrium patens*, Free‐BE showed lower editing efficiency than the conventional fused system (Fused‐BE). However, it generated fewer indels, a higher proportion of single‐nucleotide edits, and reduced out‐of‐window editing. Free‐BE also altered the spectrum of base substitutions, producing a higher proportion of C‐to‐G and C‐to‐A transversions than Fused‐BE in *P. patens* . No mutations were detected at four predicted off‐target loci examined *in P. patens*. We further validated Free‐BE in crop systems. In a transient maize protoplast reporter assay, Free‐BE achieved targeted base conversions, whereas stable transformation of wheat demonstrated successful editing of endogenous loci, although with lower efficiency than Fused‐BE. In wheat, Free‐BE similarly reduced multicytidine editing and by‐products. Together, these results establish Free‐BE as a functional modular BE strategy that broadens the plant genome‐editing toolbox and may be advantageous for applications requiring greater control over editing outcomes, despite lower overall editing efficiency.

## 1 Introduction

Although base editing (BE) technologies were rapidly validated in model plants such as *Arabidopsis thaliana* (Kang et al. 2018; Bastet et al. 2019; Ren et al. 2021; Pan et al. 2022; Jeong et al. 2025) or *Physcomitrium patens* (Veillet et al. 2020; Guyon‐Debast et al. 2021; Beraldo et al. 2023), or in crops such as maize or wheat (Zong et al. 2017; Zhang, Liu, et al. 2019; Li et al. 2020a; Han et al. 2022; Gaillochet et al. 2023; Fu et al. 2025), the production of the best allelic variants is not straightforward and may require several rounds of optimization to obtain the desired allele. Indeed, outcomes of cytidine and adenine base editors (CBEs, ABEs) are determined by an activity “window” within the R‐loop, which corresponds to the most accessible bases (Anzalone et al. 2020). As such, any cytidines or adenines present within this range can be modified, generating BE that may be unwanted (bystander products) (Kuroiwa et al. 2023). Toward more precise BE, the CBE window range can be altered by the use of various cytidine deaminase domains from different origins (Arbab et al. 2020). However, reducing the range of the BE window tends to have the disadvantage of decreasing editing efficiency (Choe and Musunuru 2021). Additional optimization can be achieved by introducing mutations in SpCas9 nickase (Molla and Yang 2019).

Most plant cytidine base editors currently rely on a fused architecture, in which the cytidine deaminase is covalently linked to the Cas9 nickase. Although this configuration generally provides high editing efficiency by ensuring continuous recruitment of the deaminase to the target R‐loop, it also has limitations. The prolonged proximity of the deaminase to the exposed single‐stranded DNA can promote editing of multiple cytidines within the activity window, increase bystander mutations, and, in some contexts, favor the formation of undesired indels. Furthermore, systematic optimization of fused editors often requires rebuilding an entire construct whenever a different deaminase, linker, or Cas9 variant is to be evaluated, making the screening of new combinations laborious, particularly in plant species where transformation remains time‐consuming.

To increase the flexibility of BE systems, several modular or split‐BE approaches have recently been proposed. Initial strategies relied on intein‐mediated protein reconstitution (Truong et al. 2015), whereas subsequent approaches used engineered recruitment systems, such as the MS2‐MCP interaction, to tether the deaminase to the Cas9 complex through modified sgRNAs (Zhang, Feng, et al. 2019; Yuan et al. 2022; Liu et al. 2025; Mu et al. 2026). These modular architectures facilitate the evaluation of alternative editing components without reconstructing complete fusion proteins. However, they still depend on engineered protein–protein or RNA–protein interactions to recruit the deaminase to the target DNA.

Here, we explored a conceptually simpler modular strategy based on the hypothesis that efficient cytidine BE could occur even in the absence of any physical linkage between the deaminase and the Cas9 nickase. In this study, the conventional editor consisting of a single fusion protein is referred to as the fused base editor (Fused‐BE), whereas the modular strategy in which the Cas9 nickase and cytidine deaminase are expressed as two independent proteins from separate plasmids is referred to as the free base editor (Free‐BE). We hypothesized that transient and independent association of the deaminase with the Cas9‐generated R‐loop would remain sufficient for BE while reducing prolonged deaminase exposure, thereby limiting unwanted by‐products such as indels, multicytidine editing, and editing outside the canonical activity window. Specifically, we compared the editing efficiency and editing outcomes of Free‐BE and Fused‐BE in the *P. patens* APT reporter system and then evaluated whether the modular strategy could be transferred to crop species using a transient reporter assay in maize protoplasts and stable endogenous genome editing in wheat. These experiments were designed to assess the trade‐off between editing efficiency and editing outcome associated with this modular architecture.

## 2 Materials and Methods

### 2.1 Plant Material and Culture

The *P. patens* Gransden strain was propagated vegetatively as previously described (Cove et al. 2009). Plants were grown on PpNH4 medium (PpNO3 medium supplemented with 2.7‐mM NH4‐tartrate) in growth chambers set at 23°C, 60% humidity, with 16 h of light (quantum irradiance of 80 μmol m^−2^ s^−1^) and 8 h of dark. Wheat cultivar Fielder and its transgenic plants were grown in the greenhouse with 16 h of light at 20°C and 8 h of dark at 15°C with constant 60% humidity. Plants were grown individually in 1‐L pots filled with soil. The maize A188 cultivar was grown in 5‐L pots filled with soil in a growth chamber in continuous dark conditions at 23°C for a 16‐h period and 18°C for an 8‐h period. Plants were harvested 14 days after sowing.

### 2.2 Plasmid Constructions

In all the expression vectors, transcripts are controlled by the maize Ubiquitin promoter (Christensen and Quail 1996), and coding sequences of editing enzymes were optimized using a monocotyledon codon bias. The plasmid pZmUbi‐BE (pBIOS12472), used in Fused‐BE experiments, expresses the fusion protein comprising a nickase version of SpCas9, nCas9 (D10A), and the cytidine deaminase CDA1 from *Petromyzon marinus* . Plasmids pZmUbi‐nCas9 (pBIOS12870) and pZmUbi‐CDA1 (pBIOS13550) expressing respectively the nCas9 (D10A) and the cytidine deaminase CDA1 were used in Free‐BE. Plasmid maps are available in Figure S1.

The *P. patens* Adenine Phosphoribosyltransferase gene (*PpAPT*) was targeted by two different sgRNAs. The sgRNA#21 is previously described in (Guyon‐Debast et al. 2021). The design of sgRNA#22 was performed using the webtool CRISPOR 5.2 (Concordet and Haeussler 2018). The expression cassette for sgRNA#22 comprises the promoter of the *P. patens* U6 snRNA (Collonnier et al. 2017), the 5′‐G‐N(19)‐3′ guide sequences targeting the *PpAPT* gene, and the tracrRNA scaffold and was synthesized by Twist Bioscience (San Francisco, California, USA). Target sequences of sgRNAs are available in Table S1.

For maize and wheat transformation constructs (Figure S2), DNA fragments were synthesized by GenScript (Piscataway, NJ) and assembled using standard Golden Gate cloning techniques (Engler et al. 2008) to create binary constructs corresponding to Fused‐BE (pBIOS12470) and Free‐BE (pBIOS13868).

### 2.3 Plant Transformation

*P. patens* protoplast transient transformation was performed as follows. Protoplasts were isolated and transformed as previously described (Charlot et al. 2022). A total of 180,000 protoplasts were transfected with 12 μg of circular plasmid DNA per transfection, in equal amounts of each co‐transfected plasmid. After transfection, the dilution of the transfection reaction, its embedding in alginate, and spreading on cellophane disks on PpNH4 medium supplemented with 0.33 M mannitol were performed as described previously (Charlot et al. 2022). Mutations in the *APT* gene confer resistance to the toxic adenine analogue 2‐fluoroadenine (2‐FA) (Fluorochem, Hadfield, UK). Regenerating protoplasts were directly selected on PpNH4 supplemented with 10 μM 2‐FA to select clones that were mutated at the *APT* locus (Collonnier et al. 2017). Growing plants were counted after 7 days and individually subcultured on fresh PpNH4 medium until harvesting for genotyping. BE rate was estimated by dividing the number of 2‐FA‐resistant plants by the number of initially regenerating plants. Three biological replicates were produced.

Maize protoplast transient transformation was performed as described previously (Wolter et al. 2017) with some modification. In brief, maize seeds from cultivar A188 were grown in soil for 2 weeks in complete darkness. Etiolated mesophyll leaves from 2‐week‐old seedlings were dissected and put directly on the surface of the digestion solution for 2–4 h at 25°C under slight shaking. Protoplast transformation was performed using 30 μg of each plasmid DNA and delivered into protoplasts by PEG‐mediated transfection. For GFP conversion analysis, maize protoplast cells were collected 48 h after transfection and gently collected by centrifugation. Supernatant was removed, and cells were resuspended in 60 μL of W5. Twenty microliters was charged into a Fuchs–Rosenthal chamber for imaging. All GFP‐positive cells were counted. BE rate was estimated by dividing the number of GFP‐positive cells by the total number of cells and corrected for transfection efficiency. Three biological replicates were produced.

Wheat plant stable transformation was performed as follows. The binary plasmids pBIOS12470 and pBIOS13868 were transformed into *Agrobacterium* EHA105, giving the strains T11782 and T12796, respectively. These strains were used to transform the wheat Fielder cultivar as described in (Ishida et al. 2015).

### 2.4 Sanger Sequence Analysis

*P. patens* genomic DNA of edited plants was extracted as previously described (Lopez‐Obando et al. 2016). Quality of genomic DNA was evaluated using primers located on a nontargeted locus, *PpRAD51‐1* gene (PpRAD51‐1#6 and PpRAD51‐1#7). Amplification of the targeted *PpAPT* locus or of the potential off‐targets for sgRNA#22 was performed using primers listed in Table S2. Sequence analysis was performed on PCR fragments by direct Sanger sequencing (Genoscreen, Lille, France).

### 2.5 NGS Analysis

DNA extractions from leaf punches (three punches of 25 mm^2^) of T_0_ wheat stably transformed plantlets (10 DAS) were performed with the NucleoMag Tissue kit (Macherey–Nagel). PCR was performed with products spanning the BE target site and carrying tails with homology to NGS adapters (Table S2). Libraries were constructed with adapters, purified with AxyPrep MAG Kit (Ampure), and quantified with the kapa Library Quantification Kit, Illumina SYBR Fast ABI Prism‐1 (Roche), and the 7900 HT Fast Real‐Time PCR System (Applied Biosystems). Next‐generation sequencing was conducted with a MiSeq Reagent Kit V2 on an Illumina NextSeq500 platform in 250 paired‐end mode.

### 2.6 Statistical Analysis

Statistical analysis was conducted using either the Kruskal–Wallis test function or Fisher's exact test in R version 4.5.0 (2025‐04‐11).

## 3 Results

### 3.1 Validation of the Free‐BE Strategy in the Moss *P. patens*

We have shown previously that BE is functional in *P. patens* (Guyon‐Debast et al. 2021) and can be used in this model plant for gene function analysis (Beraldo et al. 2023). Efficiency of editing through base editors can be easily estimated in *P. patens* by targeting the *APT* reporter gene that encodes the APRT enzyme (Figure 1a) and, as such, represents a key tool to optimize base editors. Splitting the two components of the base editor, the deaminase and the nickase, could facilitate their optimization and the testing of new versions of both modules. For this purpose, we compared in this study the BE efficiency of a “Fused Base Editor” (Fused‐BE), consisting of a single plasmid, pZmUbi‐nCas9::CDA1, which encodes a fusion of the SpCas9 nickase and the cytidine deaminase CDA1, with a BE strategy comprising two separate modules, termed the “Free Base Editor” (Free‐BE), consisting of two plasmids: pZmUbi‐nCas9, encoding the SpCas9 nickase, and pZmUbi‐CDA1, encoding CDA1 (Figure S2a). Two gRNAs, targeting the *APT* gene, whose inactivation confers resistance to the selective compound 2‐FA (Collonnier et al. 2017), were used for the comparison between Fused‐BE and Free‐BE. The editing windows of sgRNA#21 and sgRNA#22 contain 5 and 3 cytidines, respectively, allowing either the amino acids Pro75 and Pro76 or the amino acids Arg54, Tyr55, and Arg56 to be changed (Figure 1a), leading to inactivation of the APRT (Guyon‐Debast et al. 2021). Based on this, the efficiency of mutagenesis of the *APT* gene, using Fused‐BE or Free‐BE, could be easily monitored by selection on the selective compound 2‐FA of regenerating protoplasts that were transfected with either the Fused‐BE or Free‐BE system plus one of the plasmids expressing the two different gRNAs (Figure S2b). Mutation frequency expresses the frequency of edited plants among the population of regenerants (Table S3), and analysis of the *APT* gene in these plants permits the characterization of the mutations that correspond to cytidine editing accompanied or not by indels (Table S4). For the Fused‐BE, the relative efficiencies of inactivation of APRT were 7.1% using sgRNA#21 and 5.6% using sgRNA#22 (Figure 1b). The Free‐BE strategy also allows editing of the *APT* gene, although significantly less efficiently than the Fused‐BE strategy, with inactivation of the APRT at a frequency of 2.9% using sgRNA#21 and 1.9% using sgRNA#22 (for Kruskal–Wallis analysis, see Table S5). To assess the intrinsic mutagenic activity of the SpCas9 nickase, a separate experiment was performed in which the nickase was expressed alone. This resulted in extremely low mutation frequencies (0.004% using sgRNA#21 and 0.008% using sgRNA#22, Table S6), indicating that the SpCas9 nickase alone has negligible mutagenic activity. These results show that the mutagenic activity of the Free‐BE strategy is essentially due to its deaminase activity.

**FIGURE 1:**
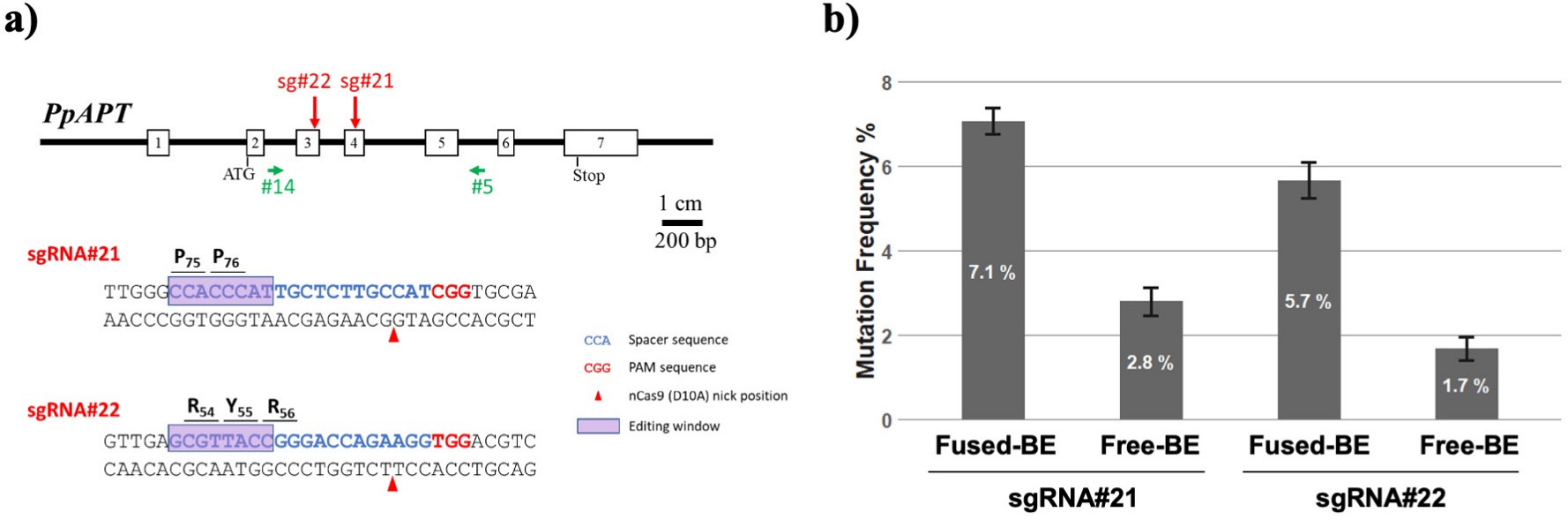
Fused and free base editing of *PpAPT* gene in moss. (a) Structure of the *APT* gene (Pp6c8_8410 from Phytozome13 dataset) and sgRNAs positions. Boxes in white represent the exons, and black lines represent the introns. The two sgRNAs positions are indicated in red, and the primers used for PCR and sequencing are shown in green. Sequence of both sgRNAs is represented, target sequence in blue, PAM in red, edition window in purple, and nickase cutting site with red arrow. Targeted amino acids with their corresponding position are indicated (P75 and P76 for sgRNA#21, R54, Y55, and R56 for sgRNA#22). (b) Mutation frequency is estimated by dividing the number of 2‐FA‐resistant plants by the number of regenerating plants. Means of three independent experiments with standard deviation. Free‐BE editing rate is significantly lower than Fused‐BE rate across sgRNAs; Kruskal–Wallis test, *p*‐value: 0.0039.

### 3.2 Quality of the Editing Is Different Between Free‐BE and Fused‐BE

As reported in various species, and as observed in this study (Table S4), cytidine editing mediated by Fused‐BE may be associated with unintended indels, also called by‐products (Komor et al. 2016). In order to estimate the quality of BE using the two different strategies, we sequenced the targeted locus in a number of *APT* gene‐mutated plants (Figure 2). As previously described for the Fused‐BE strategy (Guyon‐Debast et al. 2021), the number of indels generated increases with the number of cytidines present in the editing window and is 52.8% for sgRNA#21 compared with 7.5% for sgRNA#22. Interestingly, for the Free‐BE strategy, the number of indels is comparable using sgRNA#22 (10.8%), whereas it is decreased when using sgRNA#21 (7.1%). This decrease in by‐products (by a factor of 7) during BE cannot be solely attributed to the Free‐BE lower efficiency (by a factor of 2) compared with the Fused‐BE and could potentially be due to the activity of the deaminase itself or to its accessibility to the different cytidines present in the target sequence.

**FIGURE 2:**
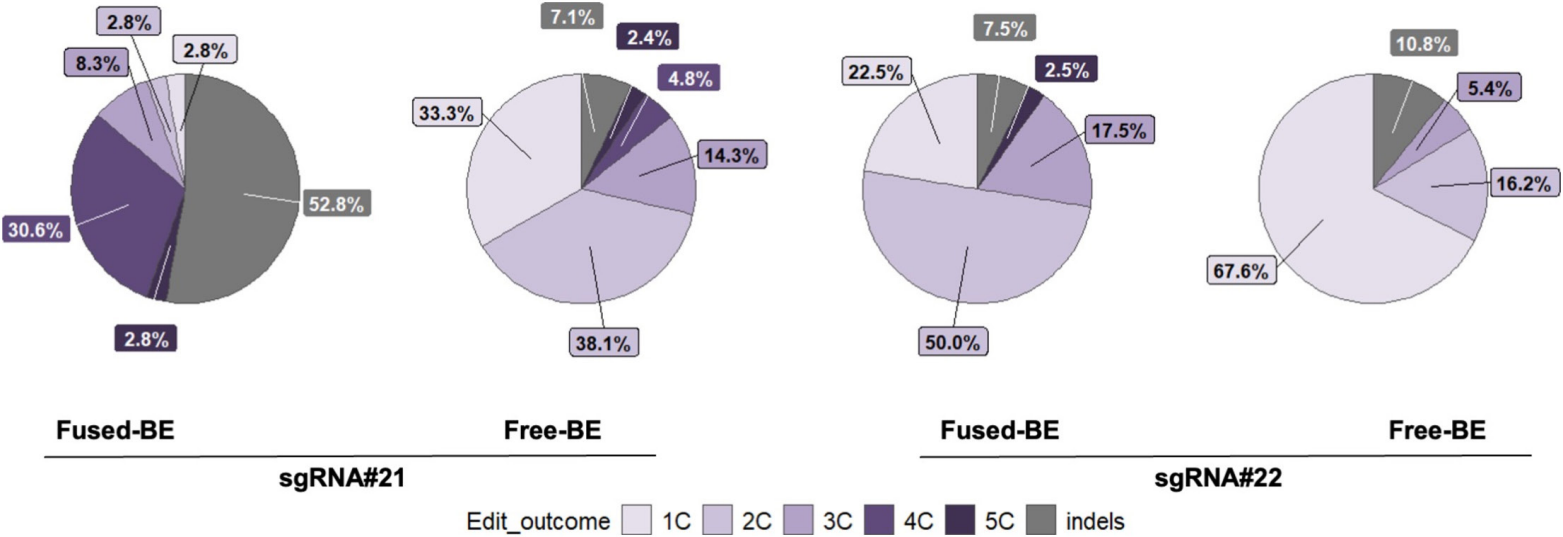
Quality of BE and pattern of editing of the *APT* gene. Proportion of plants edited for one or more (1–5) cytidines (in purple gradient) and plants that were mis‐edited (in gray), assessed by Sanger sequencing of the targeted locus for sgRNA21 and sgRNA22 using fused or free BE. The number of analyzed plants is *n* = 36, *n* = 42, *n* = 40, and *n* = 37, respectively.

The deamination of a cytidine by the cytidine deaminase CDA1 results in uracil, which has the base‐pairing properties of thymine. However, during cytidine BE in the cell, a variety of possible DNA repair outcomes determine the fate of the initial U:G product of BE, and noncanonical conversions C to G or C to A, although rare, are possible BE outcomes (Komor et al. 2016). Surprisingly, the nature of BE is different between the two BE strategies. Indeed, Free‐BE generates less C‐to‐T transitions than Fused‐BE, 69.3% and 80% respectively for sgRNA#21 and 39.5% and 76.3% respectively for sgRNA#22, and more transversions than the Fused‐BE, with a majority of C to G (Figure 3). We can thus conclude that the proportion of cytidine transversion and transition is different between Free‐BE and Fused‐BE.

**FIGURE 3:**
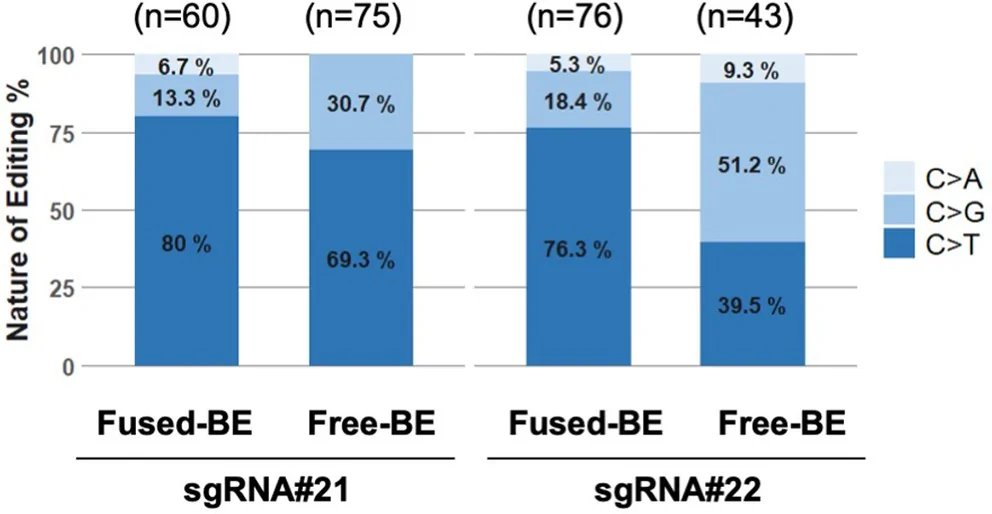
Nature of editing of the *APT* gene in *P. patens* . The nature of editing was assessed by Sanger sequencing of the targeted locus for each condition. The number of edited cytidines analyzed is *n* = 60, *n* = 75, *n* = 76, and *n* = 43, respectively.

In order to test the possible influence of the BE design on specificity, we estimated off‐target activity for both Fused‐BE and Free‐BE. For the sgRNA#22 target, no perfect 20‐bp matches were found, but four potential off‐target sequences presenting a CFD (Cutting Frequency Determination) score > 0.02 were analyzed (Haeussler et al. 2016) (Table S7). All these potential off‐target loci were amplified with surrounding primers (Table S2) and sequenced in nine plants transformed with Fused‐BE and sgRNA#22 and seven plants transformed with Free‐BE and sgRNA#22 that were all previously identified as mutated at the *APT* locus. No mutations were detected in the potential off‐target sequences for any of the tested plants.

### 3.3 Cytidines in a Given Editing Window Are Less Systematically Edited With Free‐BE Compared With Fused‐BE

In addition to indels, the extent of editing of the cytidines present in the editing window may generate unwanted editing. Extensive cytidine editing can have detrimental consequences on the expected output, as it has been shown recently in tomato, where it could alter the RNA splicing pattern of the targeted gene and result in gene knockout (Kuroiwa et al. 2023). To evaluate the quality of each BE system, we quantified the number of edited cytidines in the *APT* gene‐edited plants. For both sgRNAs, we observed that for a given edited plant, Free‐BE generates a lower number of edited cytidines compared with Fused‐BE (Figure 2). In consequence, compared with Fused‐BE, Free‐BE generates a significantly higher number of single amino acid variants (Fisher's exact test; Table S8), with 55% and 82% of the Free‐BE edited plants showing a single amino acid change for sgRNA#21 and sgRNA#22, respectively (vs. 6% and 49% with Fused‐BE).

### 3.4 Free‐BE Can Be Used for Maize Protoplast Genome Editing

To validate the functionality of the modular Free‐BE system in crops, we generated expression cassettes corresponding to the Free‐BE or Fused‐BE strategies suitable for expression in maize and wheat (Figure S2). All base editor constructs were maize codon‐optimized and cloned under the maize Ubiquitin (ZmUbi) gene promoter. We performed a transient assay in maize protoplasts to compare the functionality of the different BE systems (Fused‐BE or Free‐BE). For this purpose, we modified the BFP‐to‐GFP reporter system (Zong et al. 2017) by introducing a mutation in the BFP coding sequence that leads to a nonfluorescent BFP protein (Figure S3a), which can be corrected back to a functional fluorescent GFP by conversion of the codon CAC (His66) to TAC (Tyr66). To target the His66 codon of the BFP reporter gene, we designed an sgRNA targeting the codon CAC within the protospacer BE activity window (Figure S3a). Then, we co‐transfected maize protoplasts by PEG‐mediated transformation to introduce pZmUbi‐mBFP and pZmUBI‐BFP‐sgRNA with the Fused‐BE or Free‐BE system, respectively (Figure S3b). Using fluorescence microscopy, we evaluated the nonfunctional BFP conversion percentage to functional GFP by quantifying the GFP‐positive cell rate. As negative controls, we used the CDA1 domain alone or associated with a Cas9 nickase without any sgRNA targeting the BFP locus. We observed that those two controls yielded nonactive BFP‐to‐GFP conversion at a rate of 0.0087% and 0.0245%, respectively (Figure S3c). Transfection with the Fused‐BE yielded 1.5% of GFP‐positive cells, and using the Free‐BE system, we observed 0.6% of GFP‐positive cells (Figure S3c). These results show that our reporter system based on GFP activation through BE is functional in maize cells and suggest that the Free‐BE system is able to generate targeted BE activity in maize, although at the expense of weaker BE activity.

### 3.5 Free‐BE Can Be Used for Wheat Genome Editing

Based on the successful attempt of the Free‐BE strategy in *P. patens* and maize protoplasts, we attempted to validate the use of this strategy for multiplex BE on wheat endogenous loci using *Agrobacterium*‐mediated stable transformation to compare its efficacy to a Fused‐BE system. For Fused‐BE and Free‐BE, we selected two sgRNAs (pTaU6‐GASR7_sgRNA and pTaU6‐Lox2_sgRNA) targeting the TaGASR7 and TaLOX2 genes. These sgRNAs were selected from previously validated wheat CRISPR targets (Zhang et al. 2016) to provide well‐characterized endogenous loci for benchmarking BE performance (Figure 4 and Table S1). Both sgRNAs perfectly match the three homoeologous copies of their respective target genes. We were able to generate 77 (Free‐BE) and 291 (Fused‐BE) wheat stable T_0_ transformants. Transformed plantlets were subjected to amplicon deep sequencing analysis. Using a threshold of 20% of mutated reads, sequencing analyses revealed 33 (Free‐BE) and 235 (Fused‐BE) plants bearing at least one edited cytidine within the editing window of any of the six targeted endogenous wheat genes (Table 1). Those data confirm the functionality of the Free‐BE strategy (42.9% of edited transformants) for genome editing and, as observed in *P. patens* , the higher mutation efficiency of the Fused‐BE system (80.8% of edited transformants). Toward the goal of multiplexing BE in wheat, we evaluated the number of genes over the six possible targeted genes that were simultaneously edited for at least one cytidine (Figure 4 and Table S9). Although overall BE efficiency was weaker for Free‐BE, both BE systems were able to edit simultaneously up to five *TaGASR7* and *TaLOX2* homoeologous genes, as we found that 9.1% (Free‐BE) and 19.1% (Fused‐BE) of the edited plantlets were edited in five of six targeted genes, confirming the interest in using this strategy for multiplex editing in wheat. In the case of Fused‐BE, we could identify plants that were edited on all six targeted genes (27/235, 11.5%, Figure 4). Next, we asked whether the quality of editing would be affected using Free‐BE as compared with Fused‐BE. As previously observed in *P. patens* , both BE systems preferentially led to cytidine transition (C to T) and can also produce cytidine transversions (C to G and C to A) with an efficiency of C to A transversion up to 2.4% and 1.7%, respectively, for Fused‐BE and Free‐BE (Figure 5). Interestingly, the tendency observed in *P. patens* to produce more transversions when using the Free‐BE strategy compared with the Fused‐BE is not observed in wheat. Altogether, these results show that use of the Free‐BE system in crops allows both cytidine transitions and transversions at a similar rate to what was observed with other types of plant base editors (Mishra et al. 2020).

**FIGURE 4:**
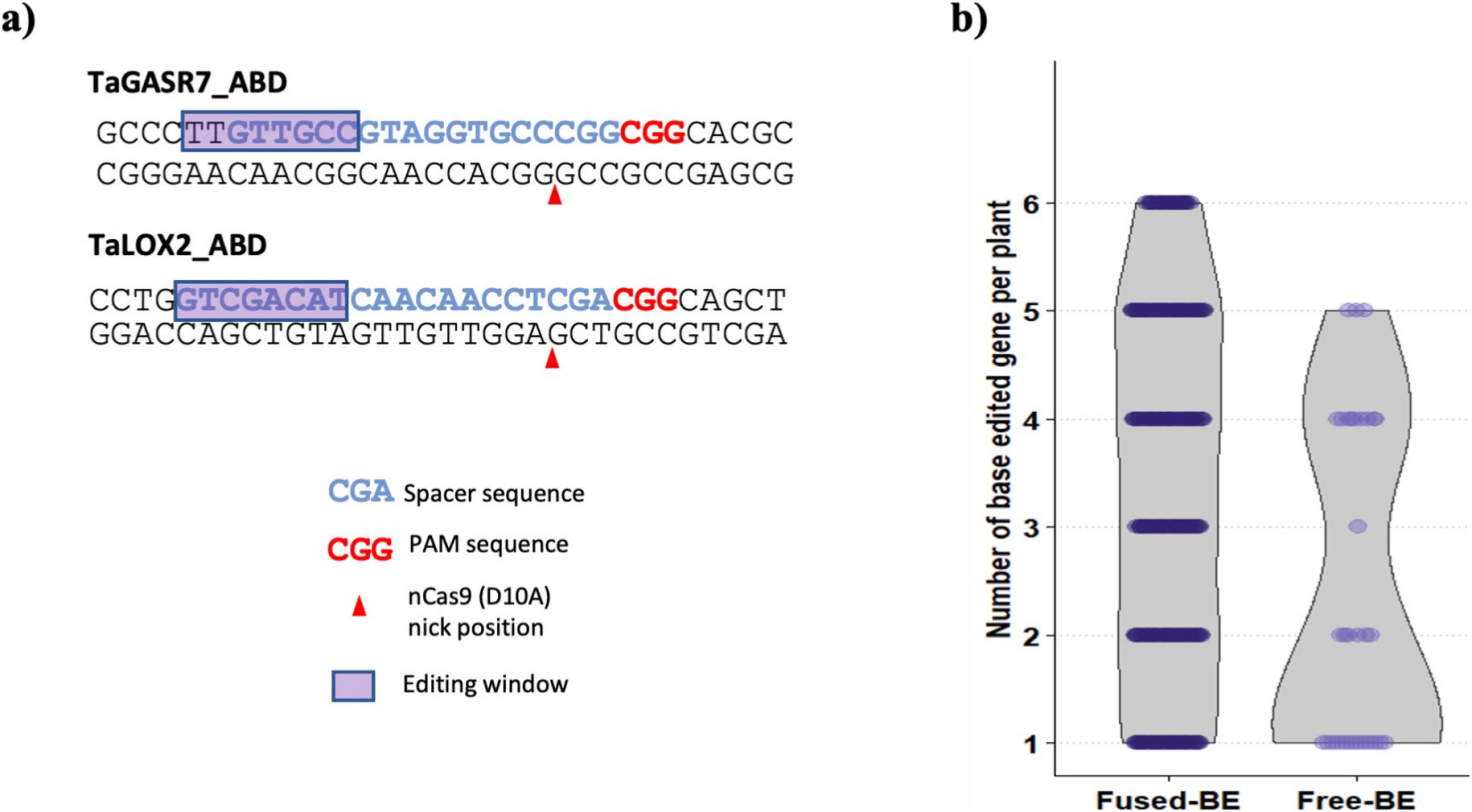
Free base editing is efficient in wheat stable plants. (a) Targeted loci within the three homoeologous *TaGASR7* genes and the three homoeologous *TaLOX2* genes and spacer positions. The sequence corresponding to the spacer is colored in blue, the PAM sequence is colored in red, the editing window is highlighted in purple, and the nickase cutting site is indicated by the red arrowhead. (b) Violin plot representing the number of edited genes per plant for each BE system; the number of dots represents the number of plants in each category between 1 and 6 edited genes per plant.

**TABLE 1:** Editing efficiency across six targeted wheat homoeologous loci.

Base editor	Nb of T_0_ plants	Nb of edited plants	Rate of edited plants (%)^a^
Fused‐BE	291	235	80.8
Free‐BE	77	33	42.9

^a^ Rate of editing expresses the frequency of edited plants for the three homoeologous TaGASR7 genes and three homoeologous TaLOX2 genes among the population of transformants.

**FIGURE 5:**
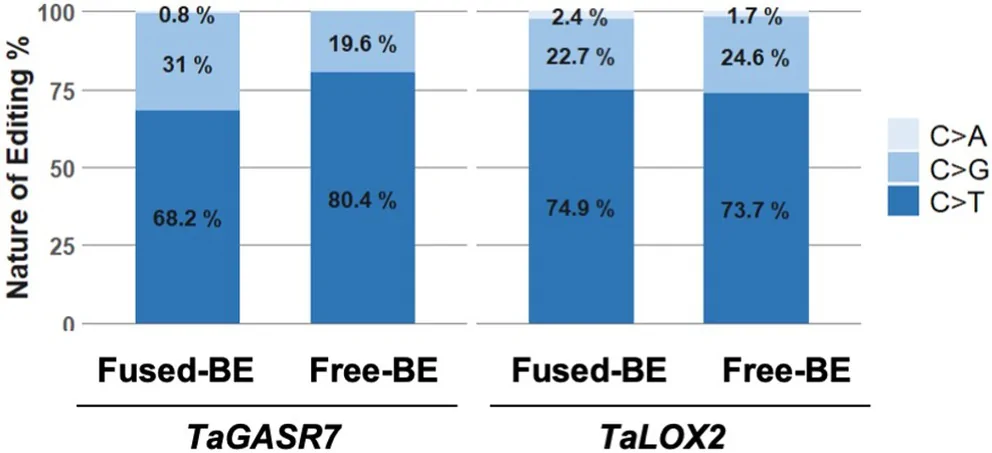
Nature of editing of the *TaGASR7* gene in wheat. Stacked bar charts showing the proportion of each possible editing outcome as a percentage of the total for each gene and BE system.

### 3.6 Multiple Cytidines Are Less Systematically Edited With Free‐BE Compared With Fused‐BE

*P. patens* experiments showed that cytidines present in the editing window were less systematically edited using Free‐BE compared with Fused‐BE. The targeted sequence of *TaGASR7* contains two cytidines next to each other, at positions C7 and C8 of the spacer sequence (Figures 6 and S4). We observed a similar proportion of plants bearing a single‐edited cytidine 52.2% and 57.7% of *TaGASR7*‐edited plants with Free‐BE and Fused‐BE, respectively. The targeted sequence of *TaLOX2* contains more cytidines compared with *TaGASR7* and is composed of seven cytidines along the spacer sequence (Figure 4). Proportions of single‐, dual‐, and triple‐edited cytidines generated by the Fused‐BE system are 21.7%, 22.6%, and 12.6%, respectively. For Free‐BE, we observed a majority of single‐edited cytidines (50%), with dual‐ or triple‐edited cytidines found at a much lower rate (10.0% and 3.8%, respectively) compared with Fused‐BE (Figure 6).

**FIGURE 6:**
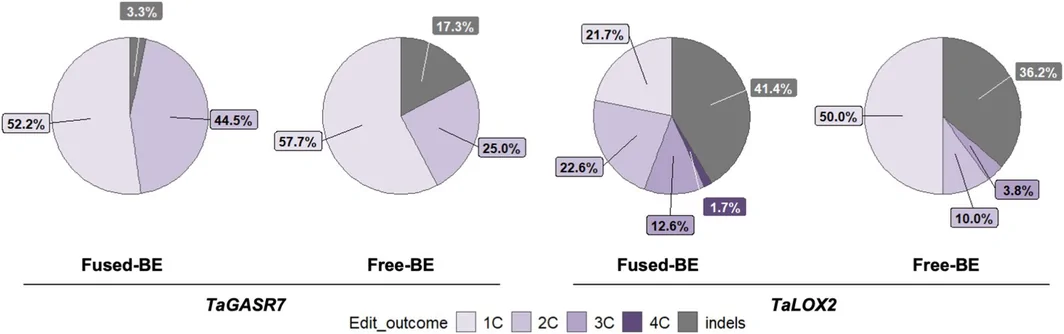
Quality of BE and pattern of editing of the *TaGASR7* and *TaLOX2* genes. Proportion (%) of plants (in purple gradient) presenting 1–5 edited cytidines within the editing window or byproducts (in gray), assessed by NGS sequencing.

In addition, when using Fused‐BE, we tend to see edited cytidines outside the predicted editing window. We isolated plants for which edited cytidines included those at position C9 and/or C5, thus much closer to the PAM (57 plants, 9.7%), or plants for which edited cytidines included those at position C21, thus outside the spacer sequence (6 plants, 1.0%, data not shown). Those types of events were not found using Free‐BE. Altogether, these results suggest that the Free‐BE system can be an interesting alternative to Fused‐BE to reach specific BE targets in crops.

## 4 Discussion

In this study, we validated a modular BE strategy, termed Free‐BE, in the moss *P. patens* and further assessed its functionality in a maize transient protoplast reporter assay and stable wheat transformants. Our findings highlight important trade‐offs between modular (Free‐BE) and fused (Fused‐BE) base editor configurations in terms of efficiency, editing precision, and the nature of resulting genomic changes.

Experiments in *P. patens* demonstrated that although both Fused‐BE and Free‐BE are capable of modifying the *APT* gene, the Fused‐BE system yielded higher BE rates for both tested gRNAs compared with Free‐BE. This is consistent with previous reports indicating robust editing efficiencies using single‐vector cytidine deaminase fusions in plants (Hillary and Ceasar 2024). However, beyond editing efficiency, we observed clear differences in the quality and fidelity of the resulting edits.

Most notably, the Free‐BE system generated fewer undesired indels than Fused‐BE, particularly with sgRNA#21 that contains a significant number of cytidines, suggesting improved control over DNA repair pathways. This reduction in by‐products, which is comparable with that obtained with the BE4 system, for example (Komor et al. 2017; Koblan et al. 2018), may reflect differences in the temporal expression or spatial distribution of the deaminase relative to the Cas9 nickase, which could affect local DNA repair outcomes.

The nature of base substitutions also diverged significantly between the two systems. Although Fused‐BE primarily induced canonical C‐to‐T transitions (78.1% for pooled sgRNAs #21 and #22), Free‐BE resulted in a lower proportion of transitions (54.4% for pooled sgRNAs #21 and #22) and a higher frequency of C‐to‐G transversions. Such altered editing spectra might derive from distinct deamination efficiencies or differing kinetics of U:G mismatch resolution in the modular system. Although they make the type of modification of the targeted amino acid(s) less predictable, they have the advantage of increasing the diversity of possible modifications. Accordingly, the improved precision observed with Free‐BE primarily reflects a reduction in by‐products, multicytidine editing, and out‐of‐window edits rather than a more predictable C‐to‐T editing outcome. Within the scope of the candidate off‐target analysis performed in *P. patens* , no unintended edits were detected at the four predicted off‐target loci examined in any of the tested lines. However, these data are limited to a small number of predicted genomic sites for a single sgRNA and do not address genome‐wide DNA off‐target activity, RNA editing, or off‐target effects in maize or wheat.

Another advantage of Free‐BE lies in its improved precision in terms of the number of cytidines that are modified in a given window. Plants edited with Free‐BE showed significantly higher rates of single cytidine edits compared with Fused‐BE (33.3% vs. 2.8% for sgRNA#21 and 50% vs. 21.7% for *TaLOX2*). Given previous findings that extensive cytidine editing can lead to gene knockouts through creation de novo of splicing sites, alteration of introns, or induction of indels in exons (Li et al. 2019; Tang et al. 2021; Kuroiwa et al. 2023), the lower incidence of multisite editing with Free‐BE may be advantageous in functional genomics and precision breeding (Li et al. 2020b). Nevertheless, this increased editing precision should be interpreted together with the lower overall editing efficiency of Free‐BE and its altered editing spectrum, which may require additional screening to recover the desired nucleotide substitution.

Importantly, we validated the Free‐BE system beyond *P. patens* , demonstrating its functionality in a maize transient reporter assay and at endogenous loci in stable wheat transformants. Although less efficient than Fused‐BE in maize protoplasts (0.6% vs. 1.5% GFP conversion), Free‐BE retained editing activity, as confirmed by a transient BFP‐to‐GFP BE reporter assay. Because this assay relies on reporter conversion in transiently transformed protoplasts rather than endogenous maize target editing, additional validation in stable maize transformants will be required to determine whether the editing characteristics observed here are maintained in the maize genome. In wheat, Free‐BE achieved base edits in 42.9% of stable T_0_ transformants, compared with 80.8% for Fused‐BE. Overall, although Free‐BE displays reduced editing efficiency relative to Fused‐BE, its improved control over editing precision and reduced production of by‐products, together with the absence of detectable edits at the limited candidate off‐target loci examined in *P. patens* , may increase the likelihood of obtaining optimal base‐edited allelic combinations in a single transformant. Its modular nature facilitates further combinatorial testing of new base editors while limiting cloning steps required to optimize construct design. Multiplex editing was also achievable: Fused‐BE enabled editing of all six targeted homoeologous loci in 11.5% of transformants, whereas Free‐BE edited up to five loci in 9.1% of plants.

Cytidine editing profiles in wheat partially recapitulated those observed in *P. patens* . Particularly, Free‐BE edited fewer cytidines per allele and maintained higher precision, avoiding editing outside the predicted activity window, unlike Fused‐BE, which caused occasional edits beyond the spacer region. Unlike in *P. patens* , however, Free‐BE did not show an increased proportion of C‐to‐G or C‐to‐A transversions in wheat, indicating that the impact of the modular architecture on editing outcomes may be species‐ or target‐dependent. These findings reinforce the potential of Free‐BE as a fine‐tuned, low‐noise editing strategy for applications requiring high target fidelity.

The characteristics of Free‐BE make it a promising alternative for applications requiring precise single‐site edits or when minimizing by‐products is critical. Future optimization of expression levels and timing of Free‐BE components could further enhance its efficiency in diverse plant systems and broaden its utility in plant genome engineering. In particular, future studies should evaluate Free‐BE at endogenous targets in stable maize transformants and perform more comprehensive assessments of DNA and RNA off‐target activity to better define its specificity across plant species.

## Author Contributions

Marie Javelle, Anouchka Guyon‐Debast, Wyatt Paul, and Fabien Nogué designed the study; Marie Javelle, Anouchka Guyon‐Debast, and Pierre‐François Perroud performed the experiments; Marie Javelle, Anouchka Guyon‐Debast, Wyatt Paul, Jean‐Philippe Pichon, and Fabien Nogué analyzed and wrote the data; Marie Javelle, Anouchka Guyon‐Debast, and Fabien Nogué wrote the manuscript. All authors read and approved the manuscript.

## Conflicts of Interest

Marie Javelle, Jean‐Philippe Pichon, and Wyatt Paul are employees of Limagrain Field Seeds. The authors declare that this affiliation did not influence the design, execution, or interpretation of the study. The remaining authors declare no conflicts of interest.

## Supporting information


**Figure S1:** Maps of plasmids used in this study. (a) Fused‐BE is encoded by the plasmid pZmUbi‐BE (pBIOS12472). (b) Free‐BE is encoded by plasmids pZmUbi‐nCas9 (pBIOS12870) and pZmUbi‐CDA1 (pBIOS13550). (c) An example of an sgRNA plasmid. Maps generated with SnapGene.
**Figure S2:** Base editing tools used in P. patens, maize, and wheat. (a) Schematic representation of the base editor pUbi‐BE for the Fused‐BE, pUbi‐nCas9 and pUbi‐CDA1 for the Free‐BE that were used in moss, maize, and wheat. (b) Schematic representation of pU6‐sgRNA constructs used for transient co‐transfection of moss protoplasts (pPpU6‐sgRNA) or maize protoplasts (pZmUBI‐BFP‐sgRNA) and for stable transformation of wheat (pTaU6‐sgRNA) and construct expressing the mutated BFP (pZmUBI‐mBFP) used as target of base editing in maize protoplast assays. Ter: terminator; HH‐RZ: hammerhead ribozyme; HDV‐RZ: hepatitis delta virus ribozyme.
**Figure S3:** Free‐BE is functional in maize protoplast transient assays. (a) Schema of mutated BFP target (top) showing the targeted sequence and the position of the cytidine that needs to be base‐edited (middle) to convert the BFP to a GFP and the predicted sequence after base editing (bottom). (b) Example of GFP‐positive cells obtained by conversion of the mutated BFP to a functional GFP observed with Zeiss Axiozoom V.16 objective ×1.0, zoom 1.2 GFP filter (scale bars: 100 μm). (c) BE rate of the GFP conversion in maize transient assays; the number of samples is indicated above each boxplot.
**Figure S4:** Editing rate per C within the editing window of the TaLOX2 genes. Using Free‐BE, we obtained a larger proportion of plants with a single‐edited cytidine within the editing window of the TaLOX2 target, as compared with Fused‐BE, which generates a large proportion of plants with two edited cytidines in the editing window.

**Table S1:** Sequences of sgRNAs used in moss, maize, and wheat, in this study.
**Table S2:** Sequences of the primers used in this study.
**Table S3:** Data of edited plants at the PpAPT locus.
**Table S4:** Possible consequences of CBE editing of the PpAPT gene using sgRNA#21.
**Table S5:** Kruskal–Wallis test on mutation frequency for each BE system and each sgRNA used in moss.
**Table S6:** Mutagenic activity of the SpCas9 nickase alone.
**Table S7:** Sequences and positions of possible off‐target sites for sgRNA22.
**Table S8:** Number of amino acid changes in edited plants (PpAPT gene).
**Table S9:** Number and rate of base‐edited wheat stable t0 plantlets showing one or several edited genes.

## Data Availability

The datasets generated and analyzed during this study are all included in the publication and Supporting Information.
