# Supplementary material for: Validation of a Modular Base Editing Strategy, Free‐BE, in Physcomitrium patens, Maize, and Wheat
Source: Plant Direct. 2026 Oct 15;10(10):e70196. doi: 10.1002/pld3.70196 (PMC13670207; doi:10.1002/pld3.70196)
Supplement: Supplementary file 1 [file PLD3-10-e70196-s001.pdf]

List of the supplementary tables:

Table S1: Sequences of sgRNAs used in moss, maize and wheat, in this study

Table S2: Sequences of the primers used in this study

Table S3: Data of edited plants at the *PpAPT* locus

Table S4: Possible consequences of CBE editing of the *PpAPT* gene using sgRNA#21

Table S5: Kruskal-Wallis Test on mutation frequency for each BE system and each sgRNA used in moss

Table S6: Mutagenic activity of the SpCas9 nickase alone

Table S7: Sequences and positions of possible off target sites for sgRNA22

Table S8: Number of amino acid changes in edited plants (*PpAPT* gene)

Table S9: Number and rate of base edited wheat stable T0 plantlets showing one or several edited genes

Table S1: Sequences of sgRNAs used in moss, maize and wheat, in this study

Used in	Name of sgRNA	Target Sequence + PAM
P. patens	sgRNA#21	CCACCCATTGCTCTTGCCAT **CGG**
	sgRNA#22	GCGTTACCGGGACCAGAAGG **TGG**
maize	BFP_sgRNA	ACCCACGGCGTGCAGTGCTT **CGG**
wheat	TaGASR7_sgRNA	GTTGCCGTAGGTGCCCGG **CGG**
	TaLOX2_sgRNA	GTCGACATCAACAACCTCGA **CGG**

Table S2: Sequences of the primers used in this study

	Name of primer	Sequences 5'-3'
Used in *P. patens*	PpRAD51.1#6	TGAGGAGGAAGTTCATCATGG
	PpRAD51.1#7	ACCGCCAATGGGTTTATGC
	PpAPT#5	ACAAGGTGGTGTCAACTTTCA
	PpAPT#14	AGATGTCGGCCTCCAAGGATG
	PpAPT#25	GTCGTCACTCTCGGTTTTG (used for Sanger sequencing)
	APTg22-OT1-F	TCGGTCAAACTTGGGGTCTC
	APTg22-OT1-R	GTGGAGTGAGAAACGGCACT
	APTg22-OT2-F	TCGGTCAAACTTGGGGTCTC
	APTg22-OT2-R	GTGGAGTGAGAAACGGCACT
	APTg22-OT3-F	AAGTTGGATTGGGCGGCATA
	APTg22-OT3-R	TGCGAAAGCAAGGTGAGCTA
	APTg22-OT4-F	TTCGTGATGTGCACGCTACT
	APTg22-OT4-R	GGCCATCCAAAGGGACTACA
Used in wheat	TaGASR7-F	CTACACGACGCTCTTCCGATCTCTTCATCCTTCAGCCATGC
	TaGASR7-R	CAGACGTGTGCTCTTCCGATCTTCCCTCCTTGGTCTTCCAG
	TaLOX2-F	CTACACGACGCTCTTCCGATCTACCACACCAGCACCATCAC
	TaLOX2-R	CAGACGTGTGCTCTTCCGATCTTGGTGGCGATGACGAAG

Table S3: Data of edited plants at the *PpAPT* locus

Name of sgRNA	Base Editor	Nb of regenerants	Nb of 2-FA[R] plants	Mutation Frequency (%) [a]	Nb of *apt* mutants analysed	Nb of edited plants	Nb of edited C	Mean of edited C/plant
sgRNA#21	Fused-BE	15143	1180	7.8	36	17	60	3.5
	Fused-BE	18619	1254	6.7				
	Fused-BE	12616	814	6.4				
	Free-BE	21225	742	3.5	42	39	75	1.9
	Free-BE	15474	424	2.7				
	Free-BE	15640	338	2.2				
sgRNA#22	Fused-BE	15971	1014	6.3	40	37	76	2.1
	Fused-BE	16798	996	5.9				
	Fused-BE	17378	820	4.7				
	Free-BE	28714	661	2.3	37	33	43	1.3
	Free-BE	11916	147	1.2				
	Free-BE	15143	225	1.5				

[a] Mutation frequency expresses the frequency of edited plants among the population of regenerants for each individual repetition.

Table S4: Possible consequences of CBE editing of the *PpAPT* gene using sgRNA#21.
PAM is in blue, nucleotide substitutions are in bold, amino acid changes are in pink, stop codons in red and deletions in orange.

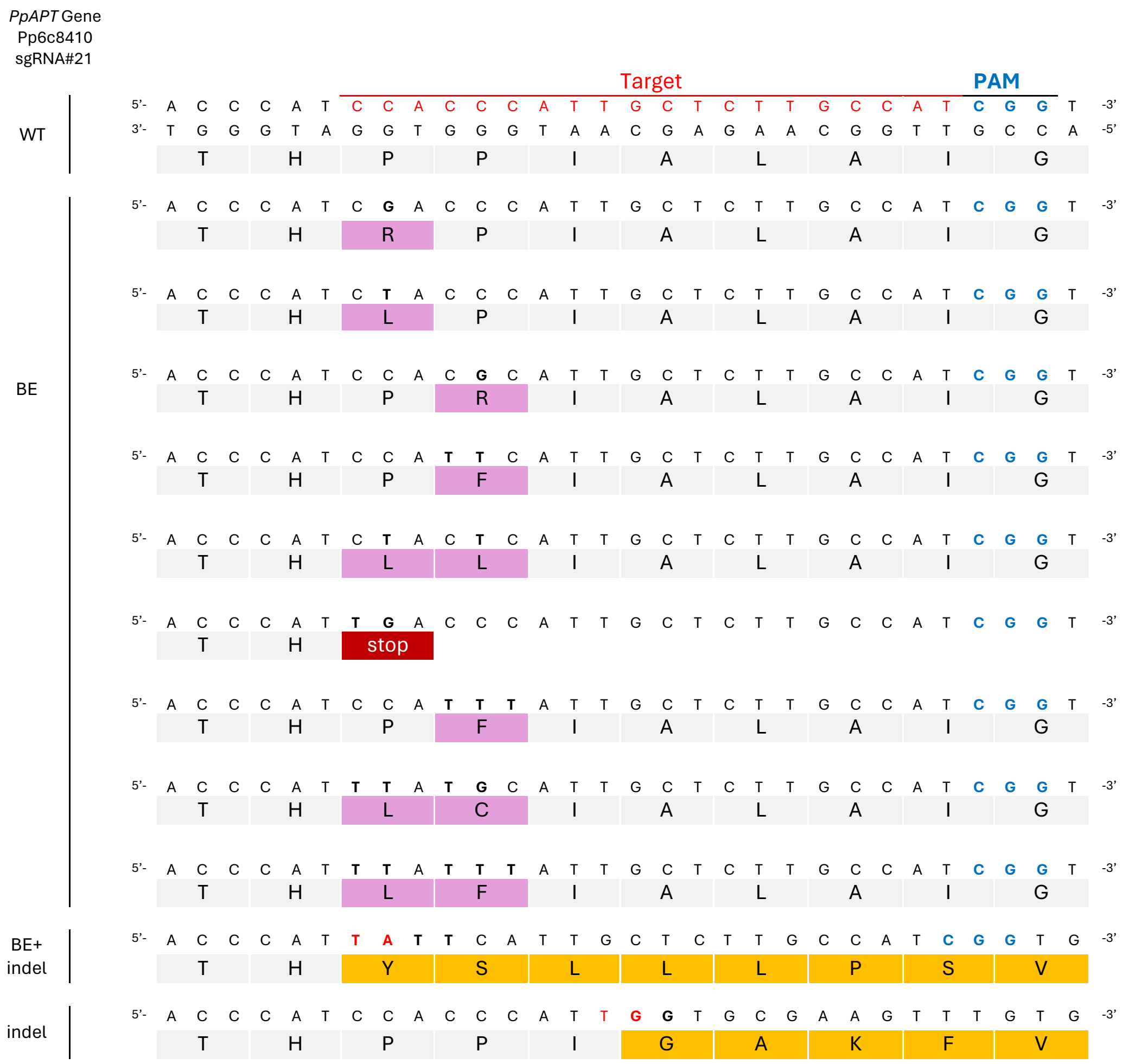

Table S5: Kruskal-Wallis Test on mutation frequency for each BE system and each sgRNA used in moss

	Data set 1	Data set 2	N1	N2	P value	significancy
Mutation frequency	Fused-BE	Free-BE	6	6	0.0039477	**
	sgRNA#21	sgRNA#22	6	6	0.2001848	ns

** indicates a P-value< 0.01 significancy level

Table S6: Mutagenic activity of the SpCas9 nickase alone

Name of sgRNA	Nb of regenerants	Nb of 2-FA^R plants	Mutation Frequency (%) [a]
PpAPT sgRNA#21	57180	1	0.002
	57594	4	0.007
	53208	2	0.004
PpAPT sgRNA#22	50809	2	0.004
	53788	8	0.015
	47168	2	0.004

[a] Mutation frequency expresses the frequency of edited plants among the population of regenerants for each individual repetition.

Table S7: Sequences and positions of possible off target sites for sgRNA22

Mismatches with the target sites are indicated in red. *P. patens* genomic off-target sequences were identified using the CRISPOR v2.0 tool (http://crispor.tefor.net).

Locus name	Target sequence + PAM	Position on *P. patens* reference genome from Phytozome[a]	CFD score[b]
sgRNA#22 Target	gcgttaccgggaccagaaggTGG	Chr08: 10812678 to 10812656	
sgRNA#22 Off-Target#1	gcgttaccaagatcagaagaTGG	Chr16: 398969 to 398991	0.394
sgRNA#22 Off-Target#2	acgatactgggaacagaaggGGG	Chr03: 23672506 to 23672528	0.144
sgRNA#22 Off-Target#3	gtgttattgggagcagaaggAGG	Chr27: 1972017 to 1972039	0.088
sgRNA#22 Off-Target#4	gcgataccagaagcagaaggAGG	Chr13: 13596455 to 13596477	0.042

[a] Coordinates of the off-target sequences were identified using Basic Local Alignment Search Tool (http://phytozome.jgi.doe.gov/pz/portal.html#!search).
[b]CFD (Cutting Frequency Determination) score given by CRISPOR (Haeussler et al. 2016). Sites with very low CFD scores < 0.02 are unlikely to be cleaved (see Haeussler et al. 2016), thus only sites with CFD score >0.02 were considered.

Table S8: Number of amino acid changes in edited plants (*PpAPT* gene)

sgRNA	Base Editor	Nb of plants with 1 aa change (%)	Nb of plants with 2 aa changes (%)	Nb of plants with 3 aa changes (%)	Nb of analyzed plants	p-value (Fisher's Exact Test)
sgRNA#21	Fused-BE	1 (6%)	16 (94%)	0	17	0.0007*
	Free-BE	21 (55%)	17 (45%)	0	38	
sgRNA#22	Fused-BE	19 (49%)	19 (49%)	1 (2%)	39	0.0215*
	Free-BE	27 (82%)	6 (18%)	0	33	

* p-value<0.05 considered as significant

Table S9: Number and rate of base edited wheat stable T0 plantlets showing one or several edited genes

Base Editor	Nb of edited genes	Nb of edited plant	Rate of edited plant (%)
Fused-BE	1	41	17.4
	2	41	17.4
	3	39	16.6
	4	42	17.9
	5	45	19.1
	6	27	11.5
Free-BE	1	14	42.4
	2	6	18.2
	3	1	3.0
	4	9	27.3
	5	3	9.1
	6	0	0

[a] Rate of editing expresses the frequency of plants with a given number of edited genes among the population of edited plants.
