# Supplementary material for: Validation of a Modular Base Editing Strategy, Free‐BE, in Physcomitrium patens, Maize, and Wheat
Source: Plant Direct. 2026 Oct 15;10(10):e70196. doi: 10.1002/pld3.70196 (PMC13670207; doi:10.1002/pld3.70196)
Supplement: Supplementary file 2 [file PLD3-10-e70196-s002.pdf]

List of the supplementary figures:

Figure S1: Maps of plasmids used in this study.

Figure S2: Base editing tools used in *P. patens*, maize and wheat.

Figure S3: Free-BE is functional in maize protoplasts transient assays.

Figure S4: Editing rate per C within the editing window of the *TaLOX2* genes.

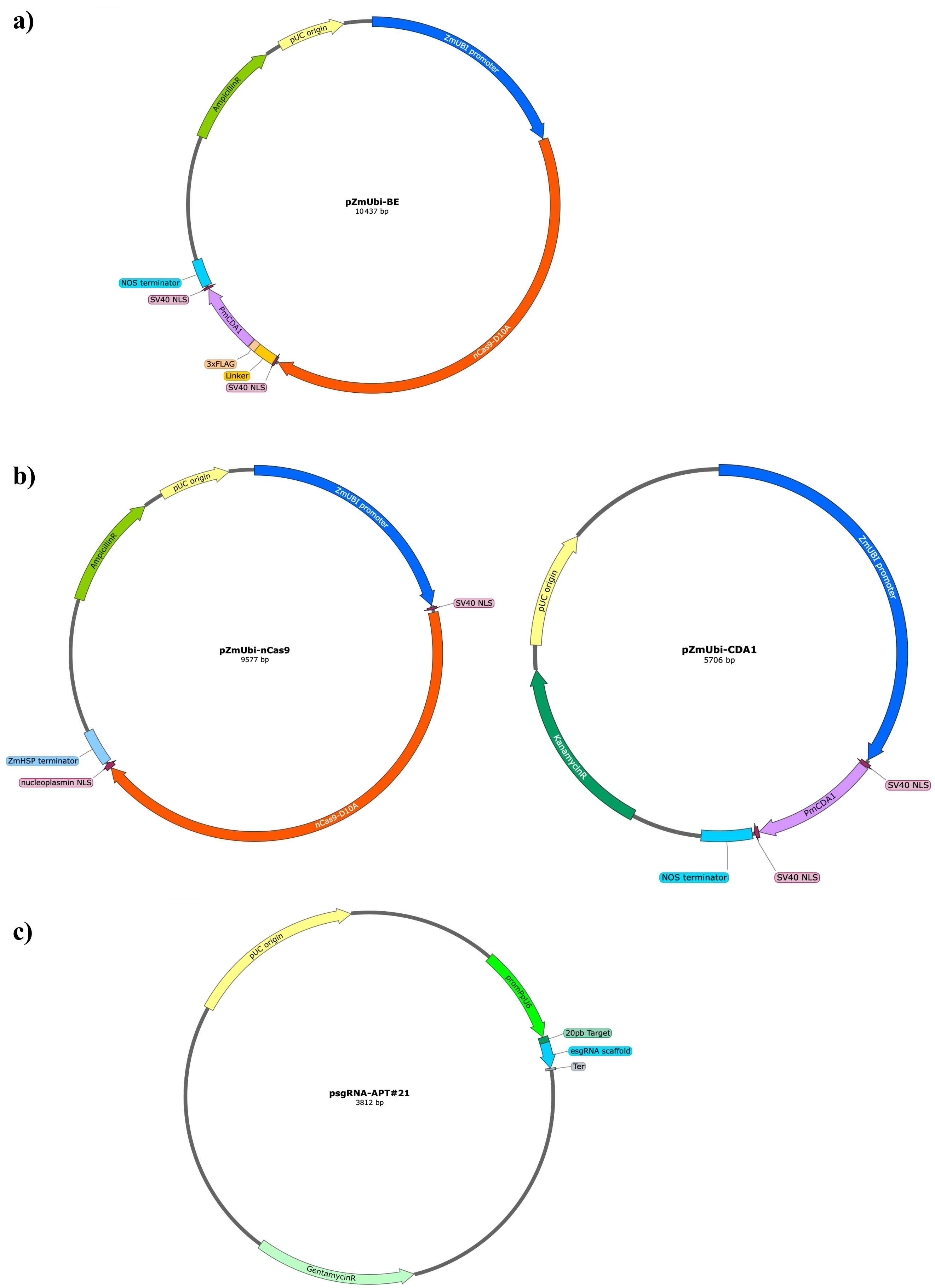

FIGURE S1: Maps of plasmids used in this study.
(a) Fused-BE is encoded by the plasmid pZmUbi-BE (pBIOS12472). (b) Free-BE is encoded by plasmids pZmUbi-nCas9 (pBIOS12870) and pZmUbi-CDA1 (pBIOS13550). (c) an exemple of sgRNA plasmid. Maps generated with Snapgene.

a)

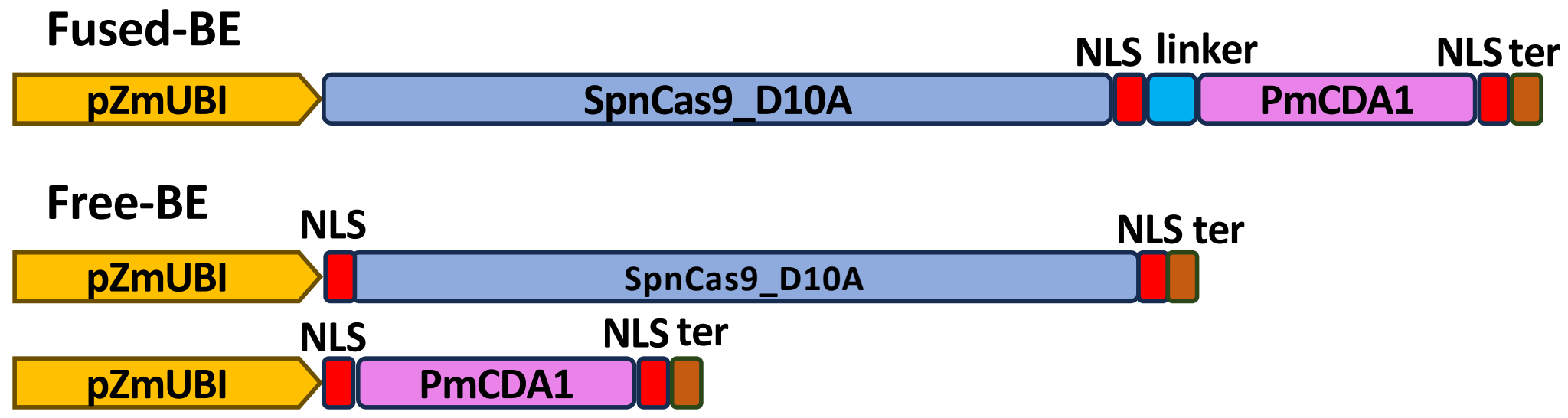

b)

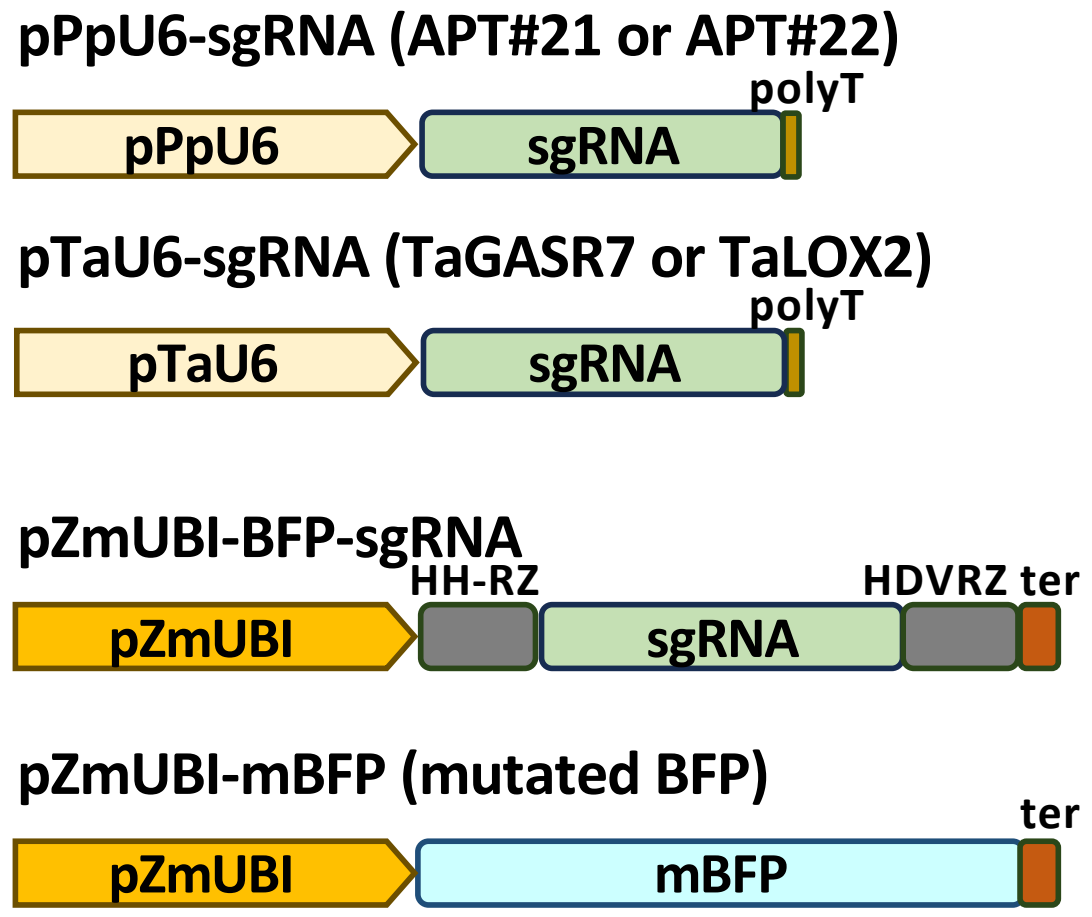

FIGURE S2: Base editing tools used in *P. patens*, maize and wheat.
(a) Schematic representation of the base editor pUbi-BE for the fused-BE, pUbi-nCas9 and pUbi-CDA1 for the Free-BE that were used in moss, maize and wheat, (b) Schematic representation of pU6-sgRNA constructs used for transient co-transfection of moss protoplasts (pPpU6-sgRNA) or maize protoplasts (pZmUBI-BFP-sgRNA) and for stable transformation of wheat (pTaU6-sgRNA) and construct expressing the mutated BFP (pZmUBI-mBFP) used as target of base editing in maize protoplast assays. Ter, terminator; HH-RZ: hammerhead ribozyme; HDV-RZ: hepatitis delta virus ribozyme.

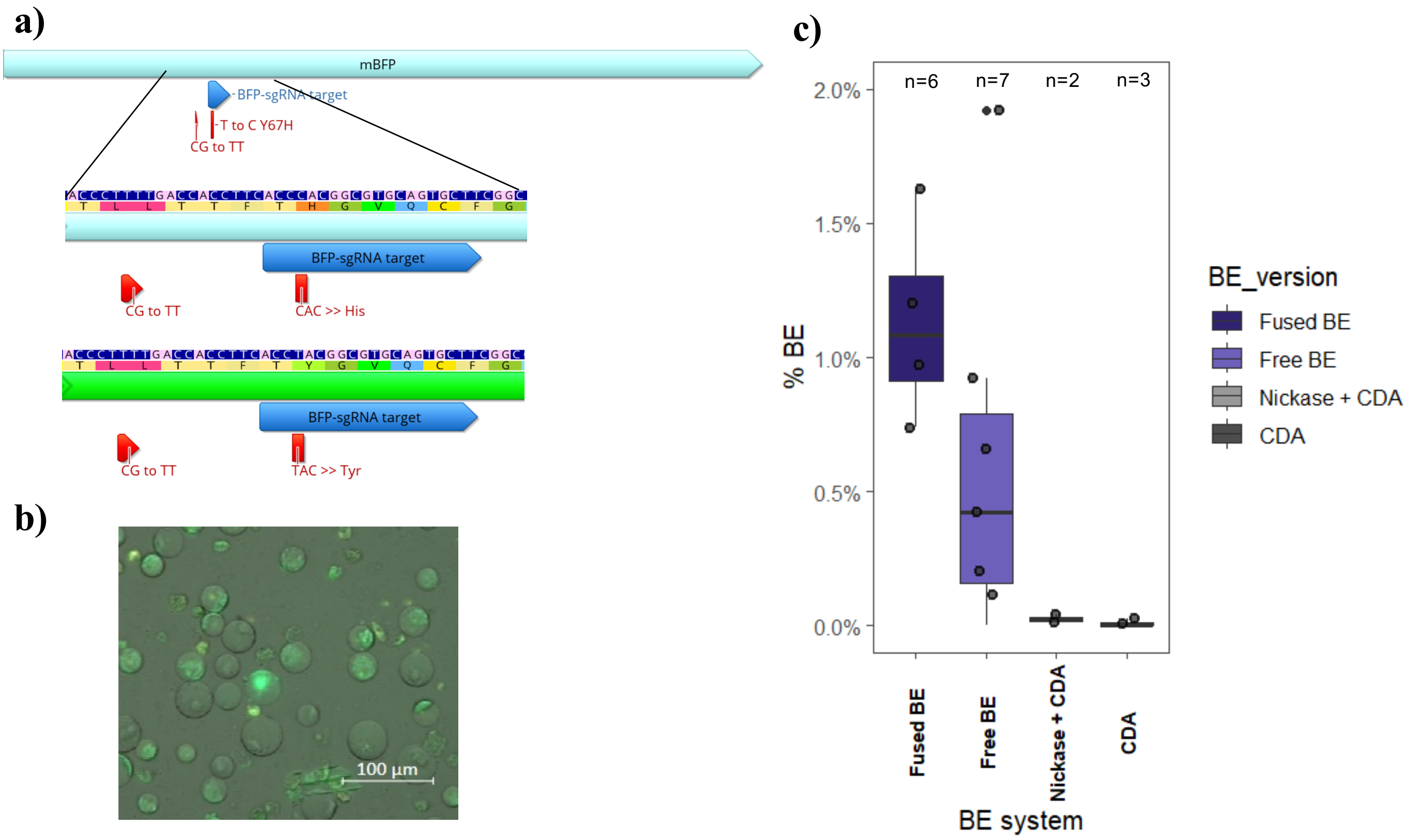

FIGURE S3: Free-BE is functional in maize protoplasts transient assays.
(a) Schema of mutated BFP target (top) showing targeted sequence and the position of the cytidine which need to be base edited (middle) to convert the BFP to a GFP and the predicted sequence after base editing (bottom). (b) Example of a GFP positive cells obtained by conversion of the mutated BFP to a function al GFP observed with Zeiss Axiozoom V.16 objectif x1,0, zoom 1,2 GFP filter (scale bars: 100 µm). (c) BE rate of the GFP conversion in maize transient assays ; the number of samples is indicated above each boxplot.

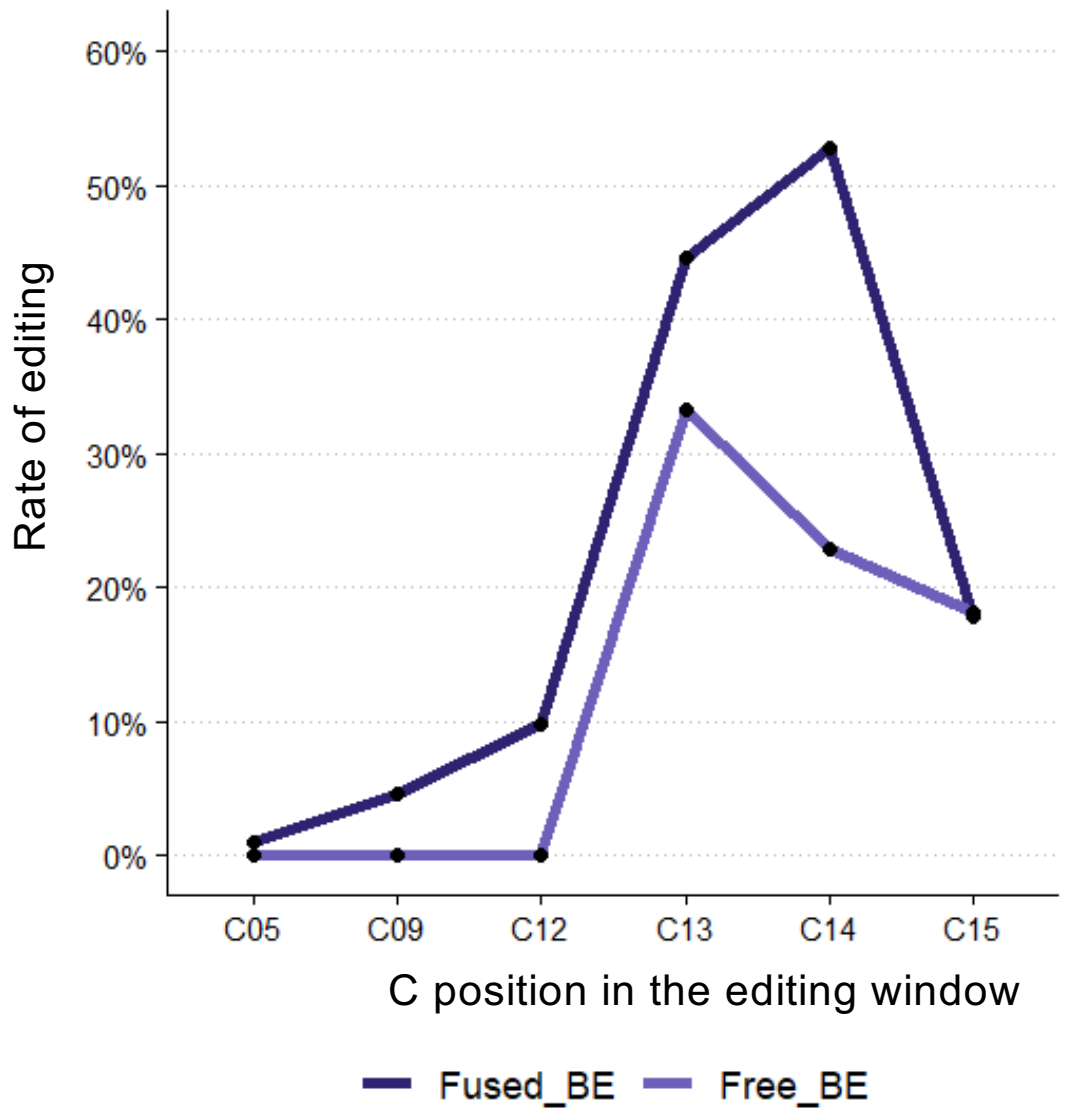

FIGURE S4: Editing rate per C within the editing window of the *TaLOX2* genes.
Using Free-BE we obtained a larger proportion of plant with a single edited cytidine within the editing window of the *TaLOX2* target, as compared to Fused-BE which generate a large proportion of plants two edited cytidines in the editing window.
